# Responses of antioxidant enzymes and key resistant substances in perennial ryegrass (*Lolium perenne* L.) to cadmium and arsenic stresses

**DOI:** 10.1186/s12870-022-03475-2

**Published:** 2022-03-25

**Authors:** Na Jiang, Zuran Li, Jingmin Yang, Yanqun Zu

**Affiliations:** 1 Faculty of Animal Science and Technology, Yunnan Agricultural University, 650201 Kunming, China; 2 College of Resources and Environment, Yunnan Agricultural University, 650201 Kunming, China; 3 College of Landscape and Horticulture, Yunnan Agricultural University, Kunming, 650201 China

**Keywords:** Antioxidant enzymes, Arsenate reductase, Compound stress, Perennial ryegrass

## Abstract

Cadmium (Cd) and arsenic (As) exist simultaneously in soil environment, which poses a serious threat to the safety of agricultural products and forage production. Four Perennial Ryegrass (*Lolium perenne* L.) cultivars with different accumulation characteristics (ʻNicaraguaʼ, ʻVenusʼ, ʻExcellentʼ and ʻMonroʼ) were selected as the material for pot experiment. The coupled responses of key components and related enzyme activities under combined stresses of Cd and As were investigated. key components contents include Non protein sulfhydryl (NPT), glutathione (GSH) and phytochelatins (PCs). The related enzyme includes (superoxide dismutase (SOD), peroxidase (POD), catalase (CAT), γ-glutamylcysteine synthetase (γ-ECS), glutathione synthetase (GSS), phytochelatin synthetases (PCSase) and arsenate reductase (AR). The results showed that Cd contents of perennial ryegrass were higher than those of As contents with TF_Cd/As_ < 1. Cd and As contents in roots were in the higher proportion than those in shoots. Compared to control, POD activities increased by 2.72 folds under 120 mg kg^−1^ As treatment. The contents of PCs increased by 5.68 folds under 120 mg kg^−1^ As treatment. Under combined Cd and As stress, the MDA contents and antioxidant enzyme activities of ʻVenusʼ were higher than those of ʻNicaraguaʼ. ʻNicaraguaʼ, a high accumulation cultivar. Under the combined stresses of Cd and As, the enzyme activities and the key components were significantly correlated (*P* < 0.05) with the contents of Cd and As. The tolerance to Cd and As was improved with increase in GSH and PCs contents and γ-ECS, GSS, PCSase and AR activities. In conclusion, the antioxidant enzyme system and key resistant substances of perennial ryegrass have important and antagonistic effects on Cd and As stresses.

## Background

Most contaminated soils contain a variety of pollutants and extremely rare for the existence of a single pollutant. Cadmium (Cd) is more toxic and contaminates in a wide range. Arsenic (As) is a metalloid, but has similar properties to heavy metals (HMs) [1]. The Cd and As co-contamination sources are accompanied by various pollution in industrial, agricultural, domestic ones and others (such as waste incineration) [2, 3]. Cd and As in plants will lead to decrease in crop yields and accumulation in humans through the food chain, thus threat human health [4]. The synergistic, independent, additive and antagonistic effects of HMs toxicity in soil–plant system have been found [5]. At present, more and more attention has been paid to soils contaminated by combined Cd and As, which has become a potential safety hazards of human and animal health. It is of important practical roles to be solved urgently.

Under HMs stress, plants produced a large number of reactive oxygen species, which denatured biomacromolecules such as proteins and nucleic acids, peroxidation of membrane lipids, and altered plant structure versus functional proteins and plant signaling [6]. Plant cells also formed resistance system to remove these reactive oxygen species (ROS). The activities of antioxidant enzymes including superoxide dismutase (SOD), peroxidase (POD) and catalase (CAT) increased to some degrees [7]. There was a certain degree of similarity between the toxicity and chelation mechanisms of Cd and As in plant cells, in which glutathione (GSH) and phytochelatins (PCs) are the key substances related to the coupled response of plants to Cd and As [8, 9]. GSH is the main non-protein mercaptan in plants and could transform to tripeptide combined Glu-Cys-Gly with catalytic of γ-glutamylcysteine synthetase (γ-ECS) and glutathione synthetase (GSS). Both Cd and As could combine with glutathione to form Cd^2+^-GS_2_ or As^3+^-GS_3_ [10]_._ Meanwhile, GSH could synthesize PCs with catalytic of phytochelatin synthetases (PCSase). HMs can chelate with Cys sulfhydryl groups within PCs and form complexes, which were transported to the vacuole for storage. PCs was involved in scavenging reactive oxygen species and attenuating toxicity. Under As stress, the synthesis of PCs may be limited by arsenate reductase (AR), which is associated with plant tolerance and sensitivity [11]. As (V) with arsenate reductase catalytic is transformed to As (III), which could be chelated with PCs and transported to vacuoles. In *Arabidopsis thalian*a, the combined expression of γ-ECS and AR can significantly improve tolerance [12]. Therefore, the expression of a series of enzyme activities and the change of the contents of key substances in the coupling process of Cd and As are worthy of further exploration.

The Perennial ryegrass (*Lolium perenne* L.) is a herbaceous plant belonging to Poaceae. It’s easy to cultivate with large biomass, rich nutrition and high economic production value. The strong resistance, wide ecological values and certain tolerance to HMs contaminated soil of Perennial ryegrass are reported, such as a certain ability to tolerate or accumulate Cu, Pb, and Zn [13]. Gholinejad et al. [14] showed that perennial ryegrass had a strong capacity to accumulate Pb and could be suitable for growth in HMs polluted soil, in which concentrations of Pb was up to 1000 mg kg^−1^. Huang et al. [15] all reported that 'aubisque', a perennial ryegrass variety from Canada, is more tolerant to Cd than 'overseederii'.

The physiological responses of different perennial ryegrass cultivars with different Cd and As accumulation characteristics has not been reported. The objective is to understand the key substances response of four perennial ryegrass cultivars to Cd and As coupling accumulation characteristics. It is expected to provide an important theoretical basis for the sustainability and remediation materials of Cd-As contaminated soil, the safe production and breeding of forage crops.

## Results

### Cd and As contents in different perennial ryegrass cultivars

The contents of Cd and As in perennial ryegrass with 3 mg kg^−1^ Cd and 120 mg kg^−1^ As treatments were significantly higher than those in control (Table 1). Cd contents in the roots of DPB, WNS, YY and ML were 82.01, 21.77, 71.02 and 49.60 mg kg^−1^, respectively, which were 167.37, 98.95, 244.90 and 198.40 times higher than those in control (*P* < 0.05). As contents were 41.53, 20.76, 18.80 and 27.14 mg kg^−1^, respectively, which were 36.75, 35.79, 9.17 and 51.21 times higher than those in control (*P* < 0.05). All of TF < 1. It is suggested that Cd and As be mostly concentrated in the root, and only minute quantity was transferred to shoot of perennial ryegrass. Under Cd and As combined stress, the TF_Cd_ of four cultivars were in order: DPB < WNS < ML < YY, and the TF_As_ are in order: YY < DPB < ML < WNS. Except that the Cd contents in DPB roots and the As contents in ML roots were the lowest under combined stress, the Cd and As contents of other cultivars were the highest under single Cd or As stress. Highly significant correlation between Cd and As contents in perennial ryegrass under combined stress were observed (*P* < 0.01, *n* = 12).

**Table 1:** Cd and As contents in perennial ryegrass under different treatments

Treatment	Cultivars	Cd contents (mg kg^−1^)			As contents (mg kg^−1^)		
		Shoot	Root	TF_Cd_	Shoot	Root	TF_As_
Control	DPB	0.43 ± 0.10 h	0.49 ± 0.11i	0.88	0.46 ± 0.10e	1.13 ± 0.06 g	0.41
	WNS	0.42 ± 0.07 h	0.22 ± 0.09i	1.91	0.00 ± 0.00e	0.58 ± 0.20 h	0.00
	YY	0.47 ± 0.06 h	0.29 ± 0.05i	1.62	0.01 ± 0.01e	2.05 ± 0.14 g	0.00
	ML	0.50 ± 0.01 h	0.25 ± 0.05i	2.00	0.00 ± 0.00e	0.53 ± 0.03 h	0.00
3 mg kg^−1^ Cd	DPB	11.91 ± 0.38d	66.33 ± 1.27e	0.18	0.00 ± 0.00e	0.92 ± 0.11 h	0.00
	WNS	9.49 ± 0.46e	66.25 ± 1.98e	0.14	0.07 ± 0.01e	1.73 ± 0.17 g	0.04
	YY	28.33 ± 0.61a	98.96 ± 1.69b	0.29	0.00 ± 0.00e	0.78 ± 0.02 h	0.00
	ML	20.16 ± 1.15c	128.04 ± 3.50a	0.16	0.00 ± 0.00e	0.68 ± 0.10 h	0.07
120 mg kg^−1^As	DPB	0.33 ± 0.06 h	1.00 ± 0.06i	0.33	5.59 ± 0.54a	51.14 ± 0.50a	0.11
	WNS	0.11 ± 0.03 h	2.76 ± 0.06hi	0.04	4.74 ± 0.20b	32.09 ± 0.66d	0.15
	YY	0.73 ± 0.15 h	2.06 ± 0.27i	0.35	2.86 ± 0.17c	34.80 ± 0.51c	0.08
	ML	0.233 ± 0.09 h	6.64 ± 0.53 h	0.04	4.16 ± 0.10b	21.10 ± 0.77f	0.20
3 mg kg^−1^Cd + 120 mg kg^−1^As	DPB	9.33 ± 0.18e	82.01 ± 3.619c	0.11	5.00 ± 0.34ab	41.53 ± 0.98b	0.12
	WNS	4.11 ± 0.20 g	27.77 ± 0.35 g	0.15	4.39 ± 0.04b	20.76 ± 1.10f	0.21
	YY	23.58 ± 1.34b	71.02 ± 0.52d	0.33	2.06 ± 0.27d	18.80 ± 0.60f	0.08
	ML	7.38 ± 0.21f	49.60 ± 0.05f	0.15	2.80 ± 0.40c	27.14 ± 0.35e	0.15

Note: Data in the table are mean ± SE; Different letters in the same column indicate significant differences at *P* < 0.05, *n* = 3. The same as below

### MDA and antioxidant enzyme activities

**Fig. 1:**
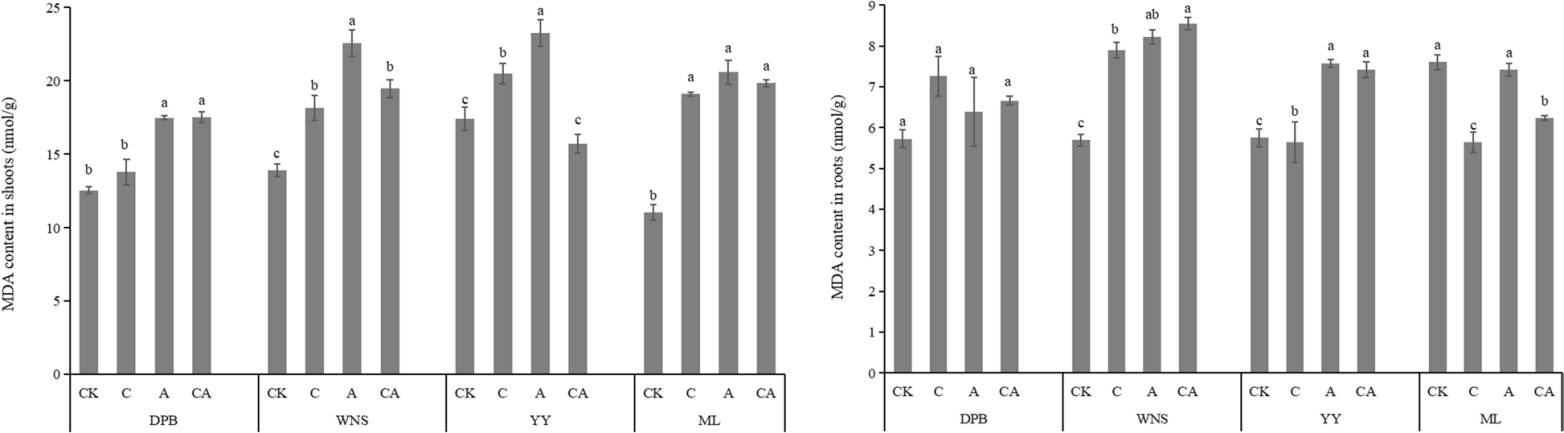
MDA contents in shoots and roots of perennial ryegrass under different treatments. CK: 0 mg kg^−1^ Cd and As (control), C: 3 mg kg^−1^ Cd, A: 120 mg kg^−1^ As, CA: 3 mg kg^−1^ Cd + 120 mg kg^−1^ As. Different letters correspond to significant differences among different treatments (*P* < 0.05). The same as below

There were some differences in MDA contents in different parts of perennial ryegrass (Fig. 1). Under control treatment, the MDA contents in the shoots of the four cultivars were 12.53, 13.87, 17.42 and 11.02 nmol g^−1^, respectively. The MDA contents in the roots were 5.73, 5.70, 5.75 and 7.61 nmol g^−1^, respectively, which showed that MDA contents in shoots were higher than those in roots. MDA contents under As and Cd stress was higher than those under control treatment. MDA contents of the shoots in ML under 120 mg kg^−1^ As treatment were the greatest, which was higher 86.83% than those under control treatment. While MDA contents of the roots in WNS under 3 mg kg^−1^ Cd + 120 mg kg^−1^ As treatment increased by 50.00% comparing to control. Under Cd and As combined treatment, MDA contents of the shoots in the four cultivars were in sequence: YY < DPB < WNS < ML, and the MDA contents of the roots were in sequence: ML < DPB < YY < WNS. Compared with other treatments, the MDA content in the four cultivars roots under 120 mg kg^−1^ As treatment were the highest. Compared with other treatments, the MDA content in the four cultivars roots under 120 mg kg^−1^ As treatment were the highest.

**Fig. 2:**
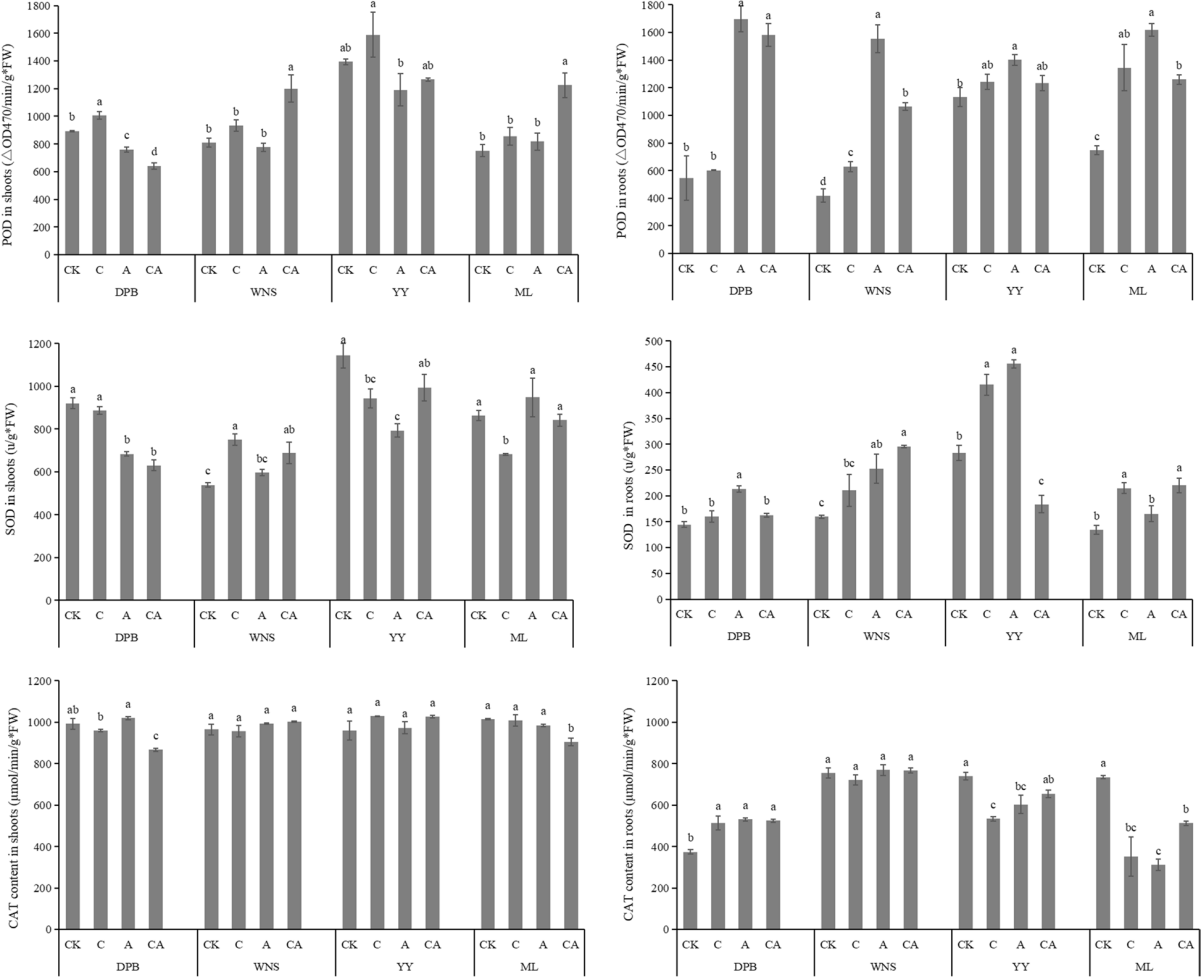
Antioxidant enzyme activities in shoots and roots of perennial ryegrass under different treatments. CK: 0 mg kg^−1^ Cd and As (control), C: 3 mg kg^−1^ Cd, A: 120 mg kg^−1^ As, CA: 3 mg kg^−1^ Cd + 120 mg kg^−1^ As. Different letters correspond to significant differences among different treatments (*P* < 0.05). The same as below

The higher POD activities in the shoots (893.33, 810.00, 1393.33 and 752.67 μmol g^−1^**)** were observed compared to roots (545.00, 418.33, 1132.67 and 747.33 μmol g^−1^**)** in the four cultivars in control treatment. POD activities of ML shoots had the greatest under 3 mg kg^−1^ Cd + 120 mg kg^−1^ As treatment, which was increased by 62.71% higher than those under control treatment. POD activities of WNS roots under 120 mg kg^−1^ As treatment was increased by 271.63% higher than those under control treatment. Among the four cultivars, POD activities of the shoots in YY were the highest under different treatments. Under Cd and As combined treatment, POD activities of the shoots in the four cultivars were in sequence: DPB < WNS < ML < YY, and the POD activities of the roots were in sequence: WNS < YY < ML < DPB. Compared with other treatments, the POD activities in both DPB and YY roots were the highest under single stress. Except for WNS and ML, the POD activities of shoots under single stress were the highest (Fig. 2).

The higher SOD activities in the shoots (920.41, 538.42, 1144.29 and 863.29 u g^−1^) were observed compared to roots (145.00, 139.81, 283.27 and 134.67 u g^−1^) in the four cultivars in control treatment. SOD activities of WNS shoots were the greatest under 3 mg kg^−1^ Cd treatment, which was 39.46% higher than those under control treatment. SOD activities of WNS roots under 3 mg kg^−1^ Cd + 120 mg kg^−1^ As treatment was 85.14% higher than those under control treatment. Under Cd and As combined treatment, SOD activities of the shoots in the four cultivars were in sequence: DPB < WNS < ML < YY, and the SOD activities of the roots were in sequence: DPB < YY < ML < WNS. Compared with other treatments, the SOD activities in DPB, WNS, ML shoots and DPB, YY roots under single stress were the highest (Fig. 2).

The higher CAT activities in the shoots (992.49, 964.64, 960.86 and 1015.16 μmol g^−1^) were observed compared to roots (374.46, 755.00, 740.37 and 735.18 μmol g^−1^) in the four cultivars in control treatment. CAT activities of WNS shoots were the greatest under 3 mg kg^−1^ Cd + 120 mg kg^−1^ As treatment, which was increased by 3.92% than those under control treatment. CAT activities of WNS roots under 120 mg kg^−1^ As treatment was increased by 41.99% compared with the control. Under Cd and As combined treatment, CAT activities of the shoots in the four cultivars were in sequence: DPB < ML < WNS < YY, and the CAT activities of the roots were in sequence: ML < DPB < YY < WNS. Compared with other treatments, the CAT activities in DPB, YY, ML shoots and DPB, WNS roots under single stress were also the higher than under Cd and As combined treatment (Fig. 2).

There was no significant correlation between POD activities and Cd or As contents under C treatment, but significant correlation between MDA, SOD and CAT activities under As stress and combined stress were observed (*P* < 0.05) (Table 2).

**Table 2:** Correlation analysis between antioxidant enzyme activities and Cd or As contents

Antioxidant enzyme	3 mg kg^−1^ Cd		120 mg kg^−1^ As		3 mg kg^−1^ Cd + 120 mg kg^−1^ As	
	Cd contents	As contents	Cd contents	As contents	Cd contents	As contents
MDA	-0.346^a^	-0.710^b^	-0.308^a^	-0.330^a^	-0.334^a^	-0.373^**b**^
POD	0.275	-0.197	0.589^b^	0.733^b^	0.585^b^	0.546^b^
SOD	-0.349^a^	-0.670^b^	-0.349^a^	-0.381^b^	-0.392^b^	-0.471^b^
CAT	-0.603^b^	-0.571^b^	-0.616^b^	-0.501^b^	-0.488^b^	-0.528^b^

Note: ^a^Indicates significant differences at *P* < 0.05 level, *n* = 48. ^b^Indicates highly significant differences at *P* < 0.01 level, *n* = 48. The same as below

### Non-enzymatic resistant substances and related enzyme activities

**Fig. 3:**
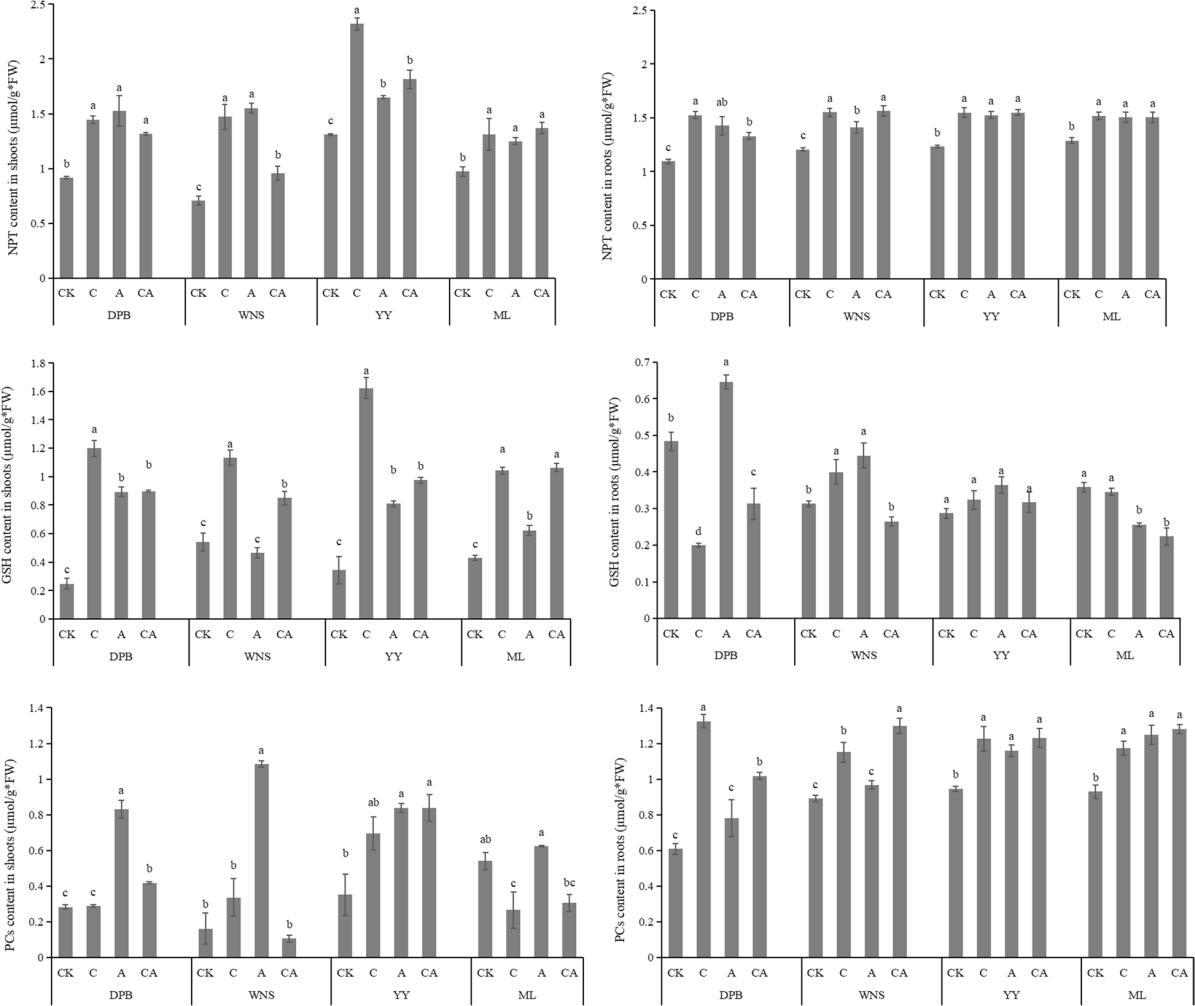
Contents of NPT, GSH and PCs in shoots and roots of perennial ryegrass under different treatments. CK: 0 mg kg^−1^ Cd and As (control), C: 3 mg kg^−1^ Cd, A: 120 mg kg^−1^ As, CA: 3 mg kg^−1^ Cd + 120 mg kg^−1^ As. Different letters correspond to significant differences among different treatments (*P* < 0.05). The same as below

The difference of NPT contents in perennial ryegrass shoots was higher than those in ryegrass roots (Fig. 3). Under control treatment, the NPT contents of shoots in the four cultivars were 0.92, 0.70, 1.31, 0.97 μmol g^−1^, and the NPT contents in roots were 1.09, 1.20, 1.23, 1.29 μmol g^−1^, respectively. NPT contents in perennial ryegrass under stress were significantly higher than those under control (*P* < 0.05). NPT contents of WNS shoots was the highest under 120 mg kg^−1^ As treatment, increased 120.13%. NPT contents of DPB roots was increased 39.40%. Under 3 mg kg^−1^ Cd + 120 mg kg^−1^ As treatment, the NPT contents in YY shoots and WNS roots were the highest. NPT contents in roots were lower than those in shoots. Except for DPB cultivars, NPT contents of ML shoots and WNS roots under single stress were higher than those under 3 mg kg^−1^ Cd + 120 mg kg^−1^ As treatment.

Under control treatment, the GSH contents in shoots of the four cultivars were 0.25, 0.54, 0.34, 0.43 μmol g^−1^, and the GSH contents in the roots were 0.48, 0.31, 0.29, 0.36 μmol g^−1^, respectively. Except for the shoots of DPB under control treatment, the GSH contents in the shoots were higher than those in the roots. In the shoots, GSH contents of DPB under 3 mg kg^−1^ Cd treatment were 383.97% higher than those under control treatment. While in the roots, GSH contents of WNS under 120 mg kg^−1^ As treatment was increased 42.25%. The GSH contents of the four cultivars shoots were different. Under 3 mg kg^−1^ Cd + 120 mg kg^−1^ As treatment, the GSH contents in ML shoots were the highest and in DPB roots were the lowest. (Fig. 3).

The PCs contents in shoots of the four cultivars were 0.28, 0.16, 0.35, 0.54 μmol g^−1^, and the PCs contents in the roots were 0.61, 0.89, 0.95, 0.93 μmol g^−1^ under control treatment, respectively, which showed that the PCs contents in the roots was higher than that in the shoots. In the shoots, PCs contents of WNS under 120 mg kg^−1^ As treatment were 567.98% higher than those under control treatment. While in the roots, PCs contents of DPB difference under 3 mg kg^−1^ Cd treatment was increased 117.22%. The PCs contents of the four cultivars shoots were most different. Under 3 mg kg^−1^ Cd + 120 mg kg^−1^ As treatment, the PCs contents in YY shoots were the highest and in WNS shoots were the lowest. However, the PCs contents in WNS roots were the highest and in DPB roots were the lowest. The PCs contents in shoots were higher than those in roots (Fig. 3).

The contents of NPT and GSH were significantly correlated with the contents of Cd and As under 3 mg kg^−1^ Cd treatment (*P* < 0.01) (Table 3). Under 120 mg kg^−1^ As treatment, NPT and GSH contents were also significantly correlated with As contents (*P* < 0.01). Under the combined stress, NPT and GSH contents were significantly positively correlated with Cd and As contents (*P* < 0.01), while PCs contents were significantly negatively correlated with Cd and As contents (*P* < 0.05).

**Table 3:** Correlation analysis between key substances, related enzyme activities and contents of As and Cd

Parameters	3 mg kg^−1^ Cd		120 mg kg^−1^ As		3 mg kg^−1^ Cd + 120 mg kg^−1^As	
	Cd contents	As contents	Cd contents	As contents	Cd contents	As contents
NPT	0.466**	0.096	0.317*	0.447**	0.528**	0.437**
GSH	0.664**	0.616**	0.483**	0.425**	0.621**	0.604**
PCs	-0.202	-0.520**	-0.328*	-0.006	-0.293*	-0.374**
γ-ECS	0.478**	-0.018	0.594**	0.656**	0.582**	0.572**
GSS	0.168	-0.300*	0.225	0.534**	0.120	0.028
PCSase	0.260	-0.136	0.120	0.076	0.605**	0.605**
AR	0.120	-0.293*	0.451**	0.633**	0.547**	0.491**

**Fig. 4:**
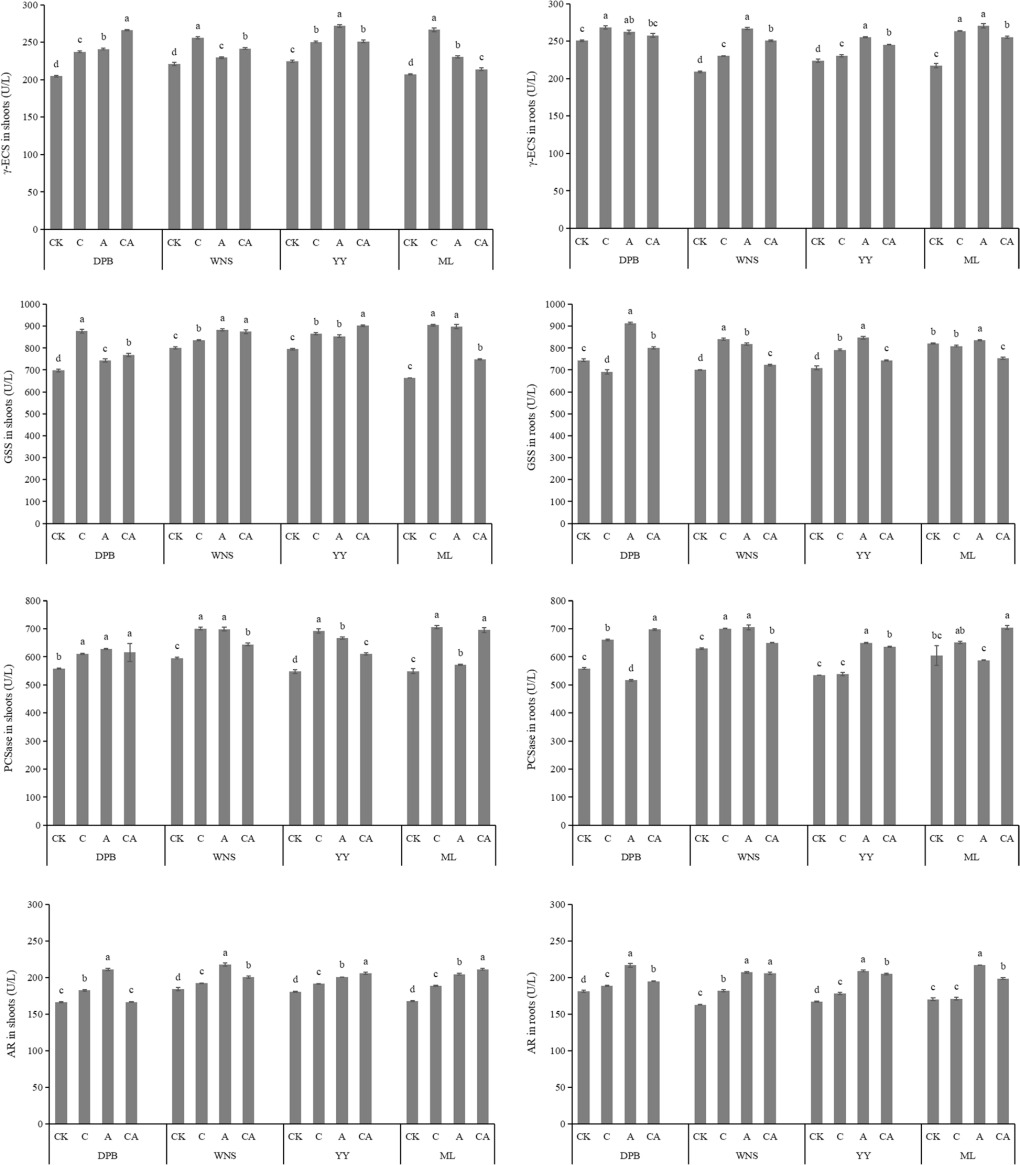
Activities of γ-ECS, PCSase, GS and AR in shoots and roots of perennial ryegrass under different treatments. CK: 0 mg kg^−1^ Cd and As (control), C: 3 mg kg^−1^ Cd, A: 120 mg kg^−1^ As, CA: 3 mg kg^−1^ Cd + 120 mg kg^−1^ As. Different letters correspond to significant differences among different treatments (*P* < 0.05)

The γ-ECS activities of the four cultivars shoots were 204.53, 220.85, 224.60 and 206.86 U L^−1^, and the γ-ECS activities of the roots were 250.61, 208.94, 223.72 and 217.32 U L^−1^ under control treatment (Fig. 4). The γ-ECS activities under 3 mg kg^−1^ Cd + 120 mg kg^−1^ As treatment were significantly higher than under control treatment (*P* < 0.05). In the shoot, the γ-ECS activities of DPB under 3 mg kg^−1^ Cd + 120 mg kg^−1^ As treatment were 30.10% higher than those under control treatment. While in the root, the γ-ECS activities of WNS under 120 mg kg^−1^ As treatment were 27.62% higher than those under control treatment. Under 3 mg kg^−1^ Cd + 120 mg kg^−1^ As treatment, the γ-ECS activities in DPB shoots and roots were the highest. However, except for DPB, the γ-ECS activities in other cultivars shoots under single stress were high.

Under control treatment, the GSS activities of the four cultivars shoots were 698.21, 801.73, 794.79 and 664.01 U L^−1^ respectively, and the GSS activities of the roots four cultivars were 744.14, 699.98, 708.89 and 820.40 U L^−1^, respectively. Except for the roots of DPB and ML, GSS activities of other cultivars roots under 3 mg kg^−1^ Cd + 120 mg kg^−1^ As treatment were significantly higher than control treatment (*P* < 0.05). In the shoots, GSS activities of ML under 3 mg kg^−1^ Cd treatment were 36.17% higher than those under control treatment. While in the roots, GSS activities of DPB under 120 mg kg^−1^ As treatment was 22.77% higher than those under control treatment. Under 3 mg kg^−1^ Cd + 120 mg kg^−1^ As treatment, the GSS activities in YY shoots were the highest and in ML shoots were the lowest. However, the GSS activities in DPB roots were the highest, and in WNS roots were the lowest. Except for the YY shoots, GSS activities of other cultivars under single stress were higher than that under 3 mg kg^−1^ Cd + 120 mg kg^−1^ As treatment (Fig. 4).

The PCSase activities in the four cultivars shoots with control treatment were 557.53, 595.40, 547.30 and 548.79 U L^−1^, and the PCSase activities of the roots were 558.22, 629.19, 533.62 and 604.35 U L^−1^. Except for DPB and ML, PCSase activities of other varieties under 3 mg kg^−1^ Cd + 120 mg kg^−1^ As treatment were significantly higher than control treatment (*P* < 0.05). In the shoots, PCSase activities of ML under 3 mg kg^−1^ Cd treatment were 38.66% higher than those under control treatment. While in the roots, PCSase activities of DPB under 3 mg kg^−1^ Cd + 120 mg kg^−1^ As treatment were 24.92% higher than those under control treatment. Under 3 mg kg^−1^ Cd + 120 mg kg^−1^ As treatment, PCSase activities in the ML shoots and roots were the highest. Except for the DPB and ML roots, PCSase activities of other cultivars under single stress were high (Fig. 4).

Under control treatment, the AR activities of the shoots in the four cultivars were 165.95, 184.25, 180.61 and 167.54 U L^−1^, respectively, and the AR activities of the roots were 181.11, 162.81, 166.87 and 170.19 U L^−1^, respectively. The activities of AR under 3 mg kg^−1^ Cd + 120 mg kg^−1^ As treatment was higher than control treatment. In the shoot, AR activities of DPB under 120 mg kg^−1^ As treatment were 27.39% higher than those under control treatment. While in the roots, AR activities of ML under 3 mg kg^−1^ Cd + 120 mg kg^−1^ As treatment were 27.59% higher than those under control treatment. Under 3 mg kg^−1^ Cd + 120 mg kg^−1^ As treatment, the AR activities in ML shoots and in WNS roots were the highest. However, the AR activities were the lowest in DPB shoots and roots. Except for YY and ML, AR activities of other cultivars under single stress were higher than those under 3 mg kg^−1^ Cd + 120 mg kg^−1^ As treatment, and were the highest under 120 mg kg^−1^ As treatment (Fig. 4).

The γ- ECS activities was significantly correlated with Cd contents under 3 mg kg^−1^ Cd treatment were observed (*P* < 0.01) (Table 3). Under As stress, γ-ECS, GSS and AR activities were highly significantly correlated with As contents (*P* < 0.01). Except for GSS, the activities of γ-ECS, PCSase and AR under combined stress were highly significantly positively correlated with Cd and As contents (*P* < 0.01).

## Discussion

### Effects of Cd-As interaction on resistance characteristics of perennial ryegrass

Soil polluted by multiple HMs formed by the simultaneous action of two or more elements. There were the toxic effects of several elements within plant tissues [16]. As a transition metal, Cd has many uses in mining, smelting, electroplating, chemical industry, electronics and even nuclear industry. As and its combined metal are commonly used in industries such as alloy smelter, agriculture, medical pharmaceuticals, and pigments, and also appear as waste residues and impurities during mineral mining. The co-contamination with Cd and As in the paddy soil is the most seriously combined pollution of toxic elements, and it is rather difficult to decrease bioavailable levels in soil because of the opposite ionic forms of bioavailable Cd (cation) and As (anion) [2]. It was reported that the different interaction of Cd, Pb and As coexisted in soil [5]. In this study, the abilities of perennial ryegrass to absorb and accumulate Cd and As under combined Cd and As stress were lower than those under single Cd or As stress, which was inconsistent with the results of Zhou’s report [17] about alfalfa. It may be that the ecological effect caused by multi-element interaction was relative to plant species, element combination, relative concentration and environmental factors. The correlation analysis showed that there was highly significant correlation between the contents of Cd and As under the combined stress (*P* < 0.01). As affected the absorption and transport of Cd of perennial ryegrass and alleviated the toxicity of Cd to a certain extent. Meanwhile, Cd also reduced the absorption of As by plants. That indicated there was antagonism between Cd and As in perennial ryegrass. It was reported that Poaceae plants contained high levels of silicon, which participated in the absorption, transport and binding of HMs and might be one of the reasons affecting the interaction between elements [18, 19]. The two elements have the same site competition, which was the direct reason for the antagonistic effect of combined pollution. These sites include active sites on the cell surface and metabolic system, such as glutathione, metallothionein, and plant chelate [20].

Under adverse conditions, the accumulation of free radicals in plants leads to the imbalance of physiological metabolism and changes in the activities of antioxidant enzymes. MDA contents, the end product of lipid peroxide, reflects the level of ROS and the degree of plasma membrane damage in plants [21]. The antioxidant enzymes activities of perennial ryegrass under combined stress were higher than those under control treatment. The roots are the first organ injured and the adaptive response, so the activities of antioxidant enzymes in the root are higher than those in shoots. However, the toxicity of HMs on roots deepened the lipid peroxidation of the cell membrane, increased the damage to the cell, and affected the synthesis of proteins and other substances in the cells. With HMs accumulated in the roots transporting to the shoot, antioxidant enzyme activities of the shoot were higher than those of the roots to maintain the metabolic balance [22]. Correlation analysis showed that MDA contents and the activities of antioxidant enzymes were significantly correlated with the contents of Cd and As, which was related to the antagonism of Cd and As. The high MDA contents under As stress indicated that perennial ryegrass should be less tolerant to As. In wheat, HMs tolerance is linked with high activity of antioxidant enzymes [23]. According to previous reports, the increase of H_2_O_2_ level will reduce the efficiency of HMs tolerance in rice, and its accumulation can be appropriately avoided through the action of oxidoreductases enzymes CAT and POD [24, 25]. The free oxygen radical (O^2−^) in perennial ryegrass was converted into low toxic H_2_O_2_ by increasing SOD activity under combined stress, while the decomposition of H_2_O_2_ into H_2_O and O_2_ with enhanced catalyzing action of POD and CAT catalyzing to protect the cells from damage [26]. Thus, high POD activity under HMs stress played a major role in in completing H_2_O_2_ removal [27]. Pan et al. [28] reported two genes *KoFSD2* and *KoCSD3* in the high Cd-tolerant *Kandelia obovata*, that showed greater SOD levels and differentially maintained the reactive oxygen mechanism when overexpressed in *Nicotiana benthamiana* under Cd toxicity. Interestingly, in this experiment, the contents of HMs in the roots of perennial ryegrass was high, but the degree of membrane lipid peroxidation of roots was lower, and the protective enzyme activity of roots in antioxidant enzyme system was generally lower than that of shoots. It shows that perennial ryegrass root has very high tolerance, and there is a strong detoxification mechanism in its root.

Non antioxidant substances and its related enzyme activities (NPT, GSH, PCs γ- ECS, GSS, PCSase and AR. NPT, GSH and PCs) were involved in the chelation of HMs ions in response to Cd and As contamination in soil [29]. Because Cd and As in plant cells mainly exist in the form of As^3+^ and Cd^2+^, they have high sulfhydryl reactivity, while sulfhydryl groups in glutathione and plant chelates could combine with As^3+^ and Cd^2+^ to form complexes, which migrate to the vacuoles of plant cells under the action of some transporters to alleviate the toxicity of plants to Cd and As [30]. GSH is a ubiquitous small molecule antioxidant in plants participating in many metabolic processes and being a scavenger of ROS induced by HMs. In this study, we the reduction of GSH contents in perennial ryegrass roots was related to more PCs synthesis in the roots. And the results confirmed that the PCs contents in roots increased. GSH also can directly complex with Cd and As to reduce toxicity so as to alleviate some toxicity in the roots [31]. Under the interaction of Cd and As, the contents of NPT and GSH in perennial ryegrass were positively correlated with the contents of Cd and As (*P* < 0.01), which showed that with the increase of Cd and As contents accumulated in perennial ryegrass, the NPT contents in vivo increased and induced GSH to synthesize PCs. As has a greater effect on the contents of PCs. GSH was catalyzed by PCSase. PCSase of perennial ryegrass showed a very significant correlation with the contents of Cd and As (*P* < 0.01). As^3+^—GS_3_ or Cd^2+^—GS_2_, which are of the high affinity substrate, could be sufficient to activate PC catalyzed by PCSase [32–34]. Under single stress, the significant relationships between GSS activity, PCs contents and As contents were observed. The activities of γ- ECS were significantly correlated with Cd and As contents under combined stress, which suggested that γ- ECS be the main reason for the high accumulation of PCs in perennial ryegrass. This was similar to the significant expression of phytochelatin, cysteine, glutathione and phytochelatin synthase in rice roots under Cd stress [35]. The activities of AR showed a highly significant correlation in the single and interaction of Cd and As (*P* < 0.01), which showed that the synthesis of PC limited by arsenate reductase activities, rather than by the synthesis ability of PC itself [10]. In *Arabidopsis*, the co-expression of γ-ECS and AR significantly improved the tolerance of As [12]. The AR of perennial ryegrass was highly expressed under As stress. The overexpression of AR led to the enhancement of As efflux and reduced As toxicity [36, 37].

NPT, GSH, PCs, γ-ECS, PCSase, and AR were the key resistance substances of perennial ryegrass in vivo Cd and As stress. Under Cd and As combined stress, a precipitation reaction might occur to form Cd_3_(AsO_4_)_2_ when both are simultaneously present in ionic form. In this study, Cd and As ions entered cells and were complexed with sulfhydryl polypeptides such as GSH and PCs contents. The more GSH and PCs to form Cd^2+^/As^3+^-PC or -GS_2_/GS_3_ complexes to achieve the detoxification of Cd and As [38]. By increasing the activities of plant chelating enzyme synthase, the co-expression of γ-ECS and AR affected the complexation of Cd and As by plant chelates in perennial ryegrass, which promoted the stronger removal of ROS induced by Cd and As and reduced the toxicity of perennial ryegrass under combined stress. This is a resistance regulation mechanism of perennial ryegrass under combined stress of Cd and As. In the future, we can use perennial ryegrass with high accumulation capacity and high tolerance to replace the difficult wild super enriched plants for soil restoration and environmental ecological restoration.

### Response of resistance characteristics of different varieties to Cd and As stress

The absorption abilities of various elements were different among cultivars with different accumulation characteristics. DPB with high Cd and high As accumulation showed strong absorption capacity under combined stress. Except that the Cd content of DPB was higher under combined stress, the Cd and As contents of other perennial ryegrass cultivars were higher under single metal treatment. Especially at the roots. It was verified that the DPB roots still has the strongest ability to absorb Cd and As under combined stress, while high Cd and low As contents in YY, low Cd and low As contents in WNS, and low Cd and high As contents in ML. However, YY had a strong transporting ability to Cd, and WNS had a strong As transporting ability.

Under combined treatment, the performance of cultivars with different accumulation characteristics was very complex. Under combined treatment, the MDA contents of WNS and ML were significantly higher than those of the control (*P* < 0.05), which suggested high degree of membrane damage and poor tolerance to As. In particular, the contents of Cd in WNS roots was low, and the degree of membrane lipid peroxidation in WNS roots was higher under combined stress, and the antioxidant enzyme activities of SOD and CAT were the highest. Therefore, WNS detoxified by antioxidant enzyme system. The MDA contents, POD and SOD activities of DPB with high Cd and As accumulation ability were less affected and shown strong tolerance. The CAT activities of DPB under As stress was quite different from those of the control, which might be other enzyme systems and HMs detoxification mechanisms in the plant joined in the scavenging effect of antioxidant system on ROS. Interestingly, as a material with high Cd, the activity of antioxidant enzymes in the roots of YY was higher than that of DPB, especially under single As stress. The degree of membrane lipid peroxidation in ML roots was higher, but the antioxidant enzyme activity was the lowest except POD activities. The detoxification ability of MLU roots to HMs was significantly lower than those of the other cultivars. The activities of antioxidant enzymes played an important protective role under combined pollution of Cd and As [39]. Zareei et al. [40] research confirmed the extent of antioxidant enzyme activity changes was cultivar dependent. Therefore, the specific reasons why different enzymes play a dominant role in roots of different cultivars need to be further studied. This suggested that the resistance mechanism of perennial ryegrass of different cultivars can not be judged only according to the change of a single resistance parameter.

The contents of NPT and GSH in the shoot of the four cultivars showed more significant differences under combined treatment. The GSH contents of DPB increased by 383.97% compared with the control under Cd stress. This might be related to the function of NPT to promote the long-distance transportation of metal ions from root to shoot. The NPT and GSH contents of DPB and YY were higher, which was similar to the research results of Li et al. [41] on different accumulation cultivars of rice under Cd stress. Because the first point of contact of the Cd and As stress is the roots. And in this experiment, the contents of HMs in roots of all cultivars was higher than those in shoots. Therefore, roots are key organ for Cd and As in perennial ryegrass. What’s more the PCs contents in the four cultivars roots were significantly higher than that of shoots. The chelating ability in roots was more stronger, which suggested that roots were the important site for perennial ryegrass to alleviate the toxicity of Cd and As to detoxification. The activities of γ-ECS, GSS, PCSase and AR of DPB and ML roots with high As accumulation ability to As were higher than those of the control, which indicated that DPB and ML alleviate the toxicity to As through the high expression of AR combined with the plant chelating protein system induced by Cd and As coupling.

DPB was more resistant to Cd and As, while WNS less resistant. The resistance parameters of perennial ryegrass cultivars with different accumulation characteristics showed obvious intraspecific differences in response to combined stress [42]. DPB had a strong ability to accumulate Cd, which was mainly chelated and detoxified by the key substances in the plant chelating protein system. High Cd and As contents increased the sulfhydryl reactivity in vivo. So the NPT contents were increased. By regulating the activities of PCSase to catalyze GSH, GSH was induced to synthesize PCs. What's more, AR participates in the transformation of As and converts pentavalent arsenic (IV) into trivalent arsenic (III). The combined expression of high γ-ECS and AR activities led to the high accumulation of PCs, which participated in the chelation of HMs ions, promoted the stronger scavenging of ROS induced by Cd and As, and reduced the toxicity of perennial ryegrass under combined stress. WNS scavenged ROS mainly by improving its antioxidant system, but its chelating ability to Cd and As was weaker than that of DPB.

Under Cd treatment, the comprehensive response index of YY and DPB were the highest, which indicated that response of perennial ryegrass to Cd showed strong resistance to adaptation (Table 4). Under As treatment, the comprehensive response index of WNS was the highest and then DPB, which showed the response of perennial ryegrass to As was sensitive. Under Cd and As combined treatment, the comprehensive response index of DPB was the highest, which showed perennial ryegrass would exist resistance response to Cd and As at the same time and detoxify to accumulation of Cd and As. Considering the indicators, GSH contents was mainly response to Cd stress, PCs contents to As stress. Under Cd and As combined stress, GSH contents was the first response and then PCs contents.

**Table 4:** Comprehensive response index (RI) of different indexes of 4 cultivars under different treatments

	3 mg kg^−1^ Cd				120 mg kg^−1^ As				3 mg kg^−1^ Cd + 120 mg kg^−1^ As			
Cultivars	DPB	WNS	YY	ML	DPB	WNS	YY	ML	DPB	WNS	YY	ML
MDA contents	36.60	69.66	15.68	47.37	51.13	107.06	65.40	84.38	56.21	90.31	19.09	62.00
POD activity	23.32	65.75	23.90	93.84	196.78	267.52	9.37	125.27	162.22	202.58	-0.16	131.31
SOD activity	6.80	71.48	29.14	38.56	21.47	68.98	30.30	33.01	-19.14	113.13	-48.21	61.02
CAT activity	44.71	-5.43	-20.73	-52.80	55.78	4.81	-17.10	-60.52	38.38	5.54	-4.55	-41.09
NPT contents	97.24	137.58	102.86	52.65	96.88	137.38	49.58	45.18	65.31	65.47	64.11	57.69
GSH contents	325.31	136.77	386.16	139.08	294.23	28.27	163.01	16.93	227.05	41.49	195.03	109.72
PCs contents	119.23	136.95	127.23	-24.66	221.82	576.47	161.00	49.16	114.93	11.06	168.18	-5.74
γ-ECS activity	22.97	25.93	14.51	50.15	22.38	31.51	34.97	35.82	32.84	29.24	21.36	20.82
GSS activity	18.60	24.12	20.58	34.75	29.21	26.93	27.04	37.07	17.85	12.38	18.55	4.40
PCSase activity	27.88	29.01	27.23	36.34	5.23	29.36	43.51	1.24	35.31	11.55	30.78	43.19
AR activity	14.04	15.97	12.90	13.13	47.03	45.26	36.39	49.63	8.01	35.29	36.74	42.90
RI	736.71	707.80	739.46	428.41	1041.94	1323.54	603.46	417.17	738.98	618.04	500.92	486.22

## Conclusion

**Fig. 5:**
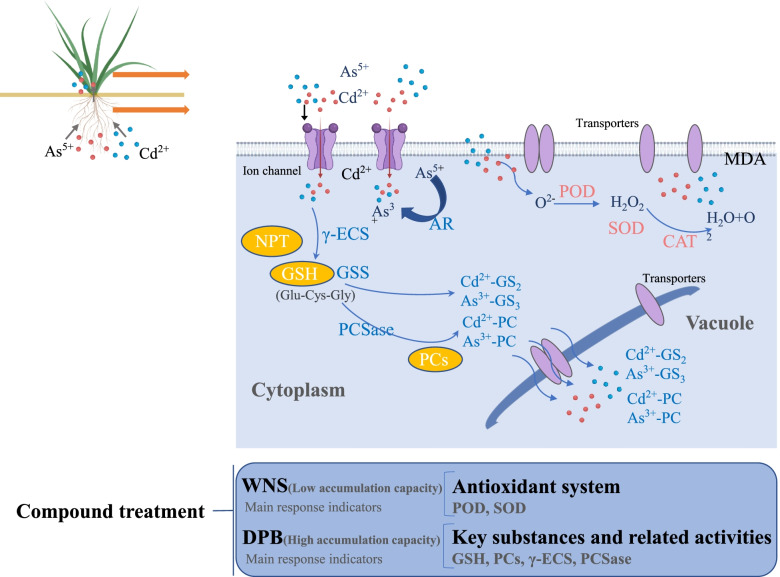
Summary graphics

In the present study, perennial ryegrass had certain resistance to Cd and As, high accumulation ability and tolerance to Cd than As.The roots was the main accumulation site for Cd and As. Cd and As stress was characterized by increased antioxidant enzyme activity, the contents of NPT, GSH and PCs, the activities of γ-ECS, GSS, PCSase and AR (Fig. 5). The degree of changes in abovementioned indicators was cultivar dependent. Nicaragua (DPB) showed not only higher accumulation capacity, but also high tolerance, it were the best in Cd and As soil pollution. It forms more substances containing sulfhydryl groups (GSH and NPT) and synthesized more PCs at the roots to complex with metals ions. Nicaragua could be recommended to plant in Cd and As soil pollution for remediation. However, this study was conducted only in pot experiments, which needs to be further verified by field experiment in the future. It is necessary to explore the related molecular mechanism for the perennial ryegrass with high accumulation ability and high tolerance.

## Methods

### Plant materials

Four cultivars (ʻNicaraguaʼ with high Cd and high As accumulation ability, ʻVenusʼ with low Cd and low As accumulation ability, ʻExcellentʼ with high Cd and low As accumulation ability and ʻMonroʼ with low Cd and high As accumulation ability) were provided by the Yunnan Agricultural University and the Beijing Zhengdao Seed Industry Co., Ltd, China. These cultivars permissions or licenses were obtained. Experimental research and feld studies on perennial ryegrass cultivars complied with China and Yunnan province local legislation. The Latin names and sources of each cultivar were shown in Table 5.

**Table 5:** The different Cd and As accumulation ability in Perennial ryegrass cultivar

Code	Cultivar	Cd	As	Latin name	Source
DPB	Nicaragua	High	High	*Lolium Perenne* L.cv. Nicaragua	American
WNS	Venus	Low	Low	*Lolium Perenne* L.cv. Venus	Denmark
YY	Excellent	High	Low	*Lolium Perenne* L.cv. Excellent	American
ML	Monroe	Low	High	*Lolium Perenne* L.cv. Monroe	Australia

The pot experiments were carried out at elevation 1937 m, N25°31′01’’ E102°75′ 33’’. The properties of soil were pH 6.18, organic matter 32.33 g kg^−1^, total nitrogen 0.90 g kg^−1^, total phosphorus 1.15 g kg^−1^, total potassium 10.02 g kg^−1^, available nitrogen 65.1 mg kg^−1^, available phosphorus 79.50 g kg^−1^, available potassium 139.19 mg kg^−1^, Cd 0.13 mg kg^−1^ and As 4.65 mg kg^−1^, respectively.

### Experimental design

Each pot (height 19.3 cm, inner diameter 25.4 cm) was filled with 3 kg soil, which was sieved through 2 mm. Based on the risk control values (Cd 3 mg kg^−1^, As 120 mg kg^−1^) in "Soil Environmental quality of Agricultural Land—Soil pollution risk Control Standard" (GB 15,618–2018), treatments were designed as follows: 0 mg kg^−1^ Cd and As (control), 3 mg kg^−1^ Cd, 120 mg kg^−1^ As and 3 mg kg^−1^ Cd + 120 mg kg^−1^ As, respectively. The exogenous Cd was prepared with CdCl_2_·2.5H_2_O and As with Na_3_AsO_4_·12H_2_O. There were three repetitions for each treatment. The seeds were sown after 15 days of soil treatment. During the growth period, the water was managed according to 60% of the water holding capacity of soil, and the plants were sampled at the 30^th^ day after germination. Parts of plant samples were dried for Cd and As contents determination. Parts of fresh plant shoots and roots were stored at 4 ℃ for physiological parameters determination.

#### Cd and As contents in plants

The plant samples, which was divided to roots and shoots, were washed with distilled water after harvesting, then put in an oven 105 ℃ for half an hour and 75 ℃ till constant weight. The samples were ground to powder and stored. Some 0.2 g samples were weighted and put into the polytetrafluoroethylene high-pressure tank. 5 ml HNO_3_ was added to overnight. The mixture solution was digested in the high-pressure tank at 145 ℃ for 4 h. After cooling, the mixture solution was constant volume and filtered. Flame atomic absorption spectrometer (Thermo ICE 3000 series) was used to determine Cd contents. Some 2 mL HCl, 3 mL thiourea and ascorbic acid solution were added in 5 mL mixture solution for 30 min, which were determined by atomic fluorescence spectrometer (Haiguang, AFS-9710) for determination of As contents [43].

#### MDA and antioxidase activities

MDA was determined with thiobarbituric acid method [44]. Some 0.5 g fresh leaves were weighted and put in a mortar. 2 mL precooled 0.05 mol L^−1^ pH 7.8 phosphate buffer were added to grind into a homogenate in an ice bath. The mortar was washed with buffer for 3 times. The mixture liquid was transfer to 5 mL centrifuge tube, and then fixed the volume to 5 mL with buffer, then centrifuged at 4500 rpm for 10 min. The supernatant was MDA extract solution. 2 mL of the extract solution was transferred into a test tube, and 3 ml 0.5% thiobarbituric acid and 5% trichloroacetic acid solution was added, then heated on the boiling water bath for 10 min. After cooling rapidly, the mixture solution was centrifuged at 4500 rpm for 10 min. The MDA contents of supernatant was determined at 532 and 600 nm by spectrophotometer (Shanghai Precision Instrument Co., Ltd., model v-5800).

POD, SOD, CAT activities were determined based on references kit method (Suzhou Grace Biotechnolgy Co., Ltd). Some 0.2 g fresh plant samples were weighed and put in a mortar, 2 mL 0.05 mol L^−1^ PBS (pH 7.5–7.8) was added to homogenize in ice bath. After centrifugation at 4 ℃ × 12,000 rpm for 10 min, the supernatant was collected and placed on ice for measurement. The POD, SOD, CAT activities was determined by ELISA Reader (Beijing Perlong New Technology Co., Ltd., DNM-9602) at 470 nm, 450 nm and 510 nm, respectively.

#### GSH, PCs and NPT contents

GSH, PCs and NPT contents were determined based on reference kit method (Suzhou Grace Biotechnolgy Co., Ltd).

NPT contents: Some 0.1 g fresh plant samples was weighted and put in a mortar, and 0.4 ml 0.1 mol L^−1^ PBS (pH 7.0) was added to homogenize in ice bath. 0.8 mL methanol was added in the mixture solution and shaken at room temperature for 10 min in order to prevent liquid evaporation loss. After centrifugation at 4 ℃ × 12,000 rpm for 10 min, the supernatant was determined the NPT contents under 412 nm of the spectrophotometer (Shanghai Precision Instrument Co., Ltd., model v-5800).

GSH contents: Some 0.1 g fresh plant samples were weighted and put in a mortar, and 1 mL 5% sulfosalicylic acid was added to homogenize in ice bath. After centrifugation at 4 ℃ × 12,000 rpm for 15 min, the supernatant was determined the contents of GSH under 412 nm with spectrophotometer(Shanghai Precision Instrument Co., Ltd., model v-5800).

[45]

$${\mathrm{PC}}_{\mathrm{s}}=\mathrm{TNP }-\mathrm{GSH}.$$

#### γ-ECS、GSS、PCSase and AR activities

Some 0.5 g fresh plant samples were weighted and liquid nitrogen added to grind into powder, and 4.5 ml 0.01 mol L^−1^ PBS (pH = 7.4) added for homogenization. After Centrifugation at 4500 rpm for 15 min, the supernatant was determinated for γ-ECS、GSS、PCSase and AR activities using γ-ECS, GSS, PCSase and AR assay kits following manufactory’s instruction (Suzhou Grace Biotechnolgy Co., Ltd) on an ELISA Reader at 450 nm, respectively. Enzymatic activity was expressed in U L^−1^.

### Statistical analysis

Translocation factor (TF) = C_shoot_ / C_root_ [46], where C_shoot_ is the contents of HMs in shoots (mg kg^−1^); C_root_ is the contents of HMs in the roots (mg kg^−1^).

[47, 48]

$$\mathrm{Response index }(\mathrm{RI})={\sum }_{i=1}^{11}\frac{{({\mathrm{indicator}}_{t})}_{i}-{({\mathrm{indicator}}_{c})}_{i}}{{({\mathrm{indicator}}_{c})}_{i}}\times 100$$

Where, RI was the comprehensive response index. Indicator_t_ and indicator_c_ expressed the data from treated groups and control groups, respectively. Indicator includes MDA contents, POD activity, SOD activity, CAT activity, NPT contents, GSH contents, PCs contents, γ-ECS activity, GSS activity, PCSase activity and AR activity when i value was respectively from 1 to 11.

Data were analyzed using descriptive statistics in Excel 2010 and plotted by origin Pro 9.1. SPSS statistics 20 statistical analysis software was used for difference significance test and correlation analysis, and Duncan test the difference of the average value of different treatments at the level of *P* < 0.05.

## Data Availability

The data that support the findings of this study are available from the first author upon reasonable request.

## Abbreviations

Cd: Cadmium
As: Arsenic
MDA: Malondialdehyde
POD: Peroxidase
SOD: Superoxide Dismutase
CAT: Catalase
GSH: Glutathione
PCs: Phytochelatins
γ-ECS: γ-Glutamylcysteine Synthetase
GS: Glutathione Synthetase
PCSase: Phytochelatin Synthetases
AR: Arsenate Reductase
